# Expectations for Engagement in Community Issues as Perceived by Young People

**DOI:** 10.34763/jmotherandchild.20212503SI.d-21-00024

**Published:** 2022-03-01

**Authors:** Katarzyna Porwit, Martyna Bójko, Magdalena Korzycka, Joanna Mazur

**Affiliations:** 1 Centre of Migration Research, University of Warsaw, Warsaw, Poland; 2 Department of Child and Adolescent Health, Institute of Mother and Child, Warsaw, Poland; 3 Department of Humanization in Medicine and Sexology, University of Zielona Gora, Zielona Gora, Poland

**Keywords:** Adolescence, participation, volunteering, developmental assets, social capital

## Abstract

**Background:**

Involvement in constructive activities is an important but rarely empirically studied developmental asset.

**Objective:**

The aim of the study is to assess the intentions of Polish youth to undertake prosocial activities after graduating from high school, and the selected determinants of these intentions.

**Material and methods:**

Anonymous surveys were conducted in 213 schools within the last round of the HBSC (Health Behaviour in School-aged Children) survey in 2018. The nationwide representative sample included 4,972 students aged 11.1–18.5 (mean age 15.4 ± 1.73). The main dependent variable was the Expectations for Engagement in Community Issues Index (EECII), built on the basis of 3 questions and ranged 0–12 points. Its relationship with 10 demographic and socio-economic variables was studied.

**Results:**

The mean EECII score was 5.59 (SD = 2.65). Seven factors and three significant two-way interactions were indicated in the general linear model. The strongest correlation was found to be between the EECII level and recognised personal values, current participation in youth groups, and the level of social ties in the neighbourhood. Girls achieve higher EECII levels than boys. However, factors such as family social position and attitudes towards school modify the gender differences. Higher family social position measured on subjective scale increases the tendency to plan prosocial activities in the group coming from low affluence families.

**Conclusions:**

Polish youth have an average inclination towards prosocial activities. When planning activities aimed at strengthening the involvement of young people, it is worth considering the specificity of various demographic and social groups.

## Introduction

For several years, intervention programs targeting children and adolescents have focused on risk factors. Currently, more attention is paid to protective factors; this trend of activities should be present in programs promoting physical, mental and social health and in preventing problem behaviours. Intervention activities may focus not only on reducing risk factors, but also on strengthening protective factors that alleviate the effects of the former. Attention is also drawn to the interactions between external determinants (such as environmental factors) and internal positive traits that work together to create overall wellbeing [1].

The theoretical basis for modern programs is often provided by the model of health assets or the model of developmental assets [2]. These approaches are aimed at understanding the factors that facilitate coping with the problems of adolescence and the challenges of the present day [3].

Health assets include factors that are assessed at three levels:

- At the individual level, these are, for example, social competences, commitment to learning, positive values, self-respect and goal orientation.
- At the community level, these are, for example, all kinds of support networks in the family, peer group and the neighbourhood.
- On the organisational or institutional level, outside the health care system, experts point to, among others, environmental resources, employment security, adequate housing conditions, democracy and social justice. Creating opportunities for participation in the society fits this rubric as well [1].

**Figure 1:**
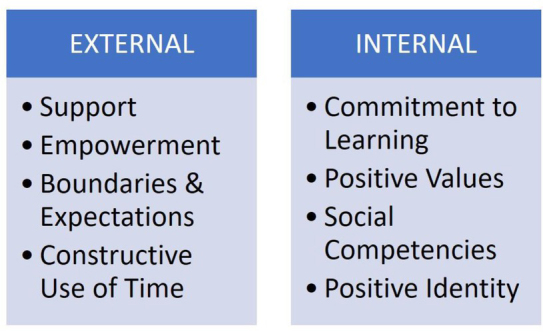
The main categories of 40 developmental assets (own elaboration based on Nakkula et al. [4])

The classification of 40 basic resources supporting proper development is based on a simpler division into external and internal factors. Both external and internal assets are divided into four main categories (Fig. 1).

The HBSC (Health Behaviour in School-aged Children) international research on health behaviours is a unique source of information that allows one to verify hypotheses about the relationship between health status and health behaviours and potential determinants. The systematically collected data also make it possible to assess what percentage of young people have adequate health-enhancing resources.

In the last round of this research, three interesting additional packages related to the Lerner theory of positive development (PYD, Positive Youth Development) were proposed. The PYD promotes a strength-based approach rather than a deficit-based approach [5]. The HBSC protocol question packages concerned mental health enhancement by active engagement, social self-efficacy and sense of unity [6]. In Poland, all three packages were linguistically adapted and tested in pilot studies, but only the first two were included in the mainstream survey. So far, publications based on the 2018 Polish HBSC results have used the social self-efficacy (SSE) scale, showing results relating to four age groups, the SSE as a health outcome and the SSE as a determinant of health [7]. However, there are no studies on active engagement. The questions in the HBSC protocol touch upon issues such as volunteering and wider conscious participation in society. Active participation may be treated as an individual health asset, but its level indirectly proves institutional and organisational conditions conducive to prosocial activity.

Wong et al. [8] presented various models of young people’s participation in the life of the local community, starting from a simple division based on Arnstein's concept (non-participation, tokenism, citizen power) and the classic model of Hart's participation ladder further developed by Shier.

In line with the above assumptions, participation may be considered in two dimensions. On the one hand, it is the progress of commitment expressed through the successive levels: (1) openings, (2) opportunities and (3) obligations. On the other hand, the involvement of young people is expressed through the level of autonomy in decision-making.

In the scoping review by Hernantes et al. [9], papers were identified describing the importance of volunteering for the proper development of adolescents and young adults. The most interesting works selected as a result of the review were assigned to the PYD theory and its five components, known as the five “Cs” (competence, confidence, connection, character and caring). Most of the articles were from the U.S. and concerned people ages 12–24. It can be assumed that different countries and cultures have different cultural approaches to volunteering and different legal and organisational conditions. The authors of this review concluded in the summary that it is necessary to undertake research into the importance of volunteering in various populations and in a changing reality.

Under Polish conditions at the organisational level the most available form of voluntary service for young people is that organised by schools. Education law stipulates that ‘the system of education ensures formation of prosocial attitudes among students, among others by creating opportunities to participate in voluntary service which promotes active involvement of students in social life’1Education Law Act, Ustawa z dnia 14 grudnia 2016 r. Prawo oświatowe, (Dz. U. z 2021 r. poz. 1082) . On this basis the school statute describes the methods of organising and implementing the school’s voluntary activities. Voluntary services are also increasingly included in schools’ preventive and educational programs as an important protective factor. Volunteer activities at the school level may be initiated by teachers as well as students. A voluntary services board may be appointed in a school and work under the care and supervision of a selected teacher. All forms of voluntary service, those that are associated with schools and those that are not, as well as the rights of volunteers, are regulated by the act on public utility activities and voluntary service2Act on Public Benefit Activity and Volunteerism, Ustawa z dnia 24 kwietnia 2003 r. o działalności pożytku publicznego i o wolontariacie (Dz. U. z 2020 r. poz. 1057, z 2021 r. poz. 1038, 1243, 1535.).

In light of the available knowledge, there is no empirical research on contemporary Polish youth. The work presented below is the first publication using the 2018 HBSC results on the level of social involvement of young people and the approach to volunteering.

## Objective

The aim of the research is to assess the occurrence of intentions of prosocial activities among Polish youth and selected determinants of these intentions.

The following research questions were formulated:

- To what extent are Polish students interested in taking future actions for the benefit of the community in which they live?
- Which factors are most conducive to such intentions?

## Material and methods

### Survey participants and research organisation

The study was carried out as part of the last round of the HBSC international research on health behaviour of school children in the 2017/2018 school year. These were anonymous surveys conducted with the use of the auditorium method in schools, using a paper questionnaire. Young people from three school grades were qualified for the analysis according to the transitional education system in force in that year (7th grade of primary school, 3rd grade of junior secondary school and 2nd grade of general and technical secondary schools). The mean age of the respondents was 15.4 years (SD = 1.73), the age range was 11.1 to 18.5 years. The criterion for inclusion in the analyses was to complete a short block of questions about the intentions of prosocial activities, described later. This way, the 11-year-olds covered by the HBSC survey, who completed a much shorter questionnaire, were eliminated. The study sample consisted of 4,972 students, including 2,220 boys (44.7%) and 2,752 girls (55.3%). In the following school grades there were 1,835, 1,697 and 1,440 students. The share of young people living in urban and rural areas was proportional to the national degree of urbanisation (39.5% of rural population, 60.5% of urban population, including 25.8% living in cities with more than 100,000 inhabitants). The research was carried out in 213 randomly selected schools located in all 16 provinces and in 114 districts. The districts were randomly selected, stratified according to the local level of deprivation [10], and the number of respondents was proportional to the number of people in the subsequent deciles of the local deprivation index. More detailed information on the research organisation is contained in two key national reports [11, 12]. The study design and the thematic scope of the questionnaire received a positive opinion from the Bioethics Committee at the Institute of Mother and Child in Warsaw (opinion no. 17/2017 of March 30, 2017).

### Tools and indicators

Most of the variables and scales analysed were derived from the international HBSC research protocol. This protocol includes mandatory questions, used in all member countries of the network participating in each round of research, as well as optional questions, used in selected countries. The two visual scales (school achievement and family social position) described below were included in the Polish questionnaire as additional national items. Also, the question about the place of residence is traditionally attached to the Polish questionnaire outside the international protocol.

### Dependent variable

The main dependent variable is the Expectations for Engagement in Community Issues (EECII) index, built based on three items. Students rated on a scale of 1 (completely unlikely) to 5 (very likely) assessed how likely it was that they would engage in the following activities after finishing high school:

- Volunteering to help people in need.
- Engaging in matters that affect their community, such as health and safety.
- Working in a group to solve a problem in the community they live in.

These questions were proposed by the Mental Health and Wellbeing working group within the HBSC network based on a literature review [13]. The questions belong to the *Positive mental health through active youth engagement* question package, which also includes the second active engagement package, from which one question was taken about the current participation in youth groups.

The EECII index ranges from 0–12 points. In the examined Polish sample, this scale is unidimensional according to the principal components analysis (PCA) with reliability at the Cronbach’s alpha level = 0.676.

### Independent variables

When examining the diversity of the EECII index in various groups of Polish adolescents, demographic characteristics (gender, age, place of residence) and seven selected individual and socio-economic conditions were considered. Three age groups (13, 15, 17 years), corresponding to the school grades mentioned above, were distinguished. The place of residence was coded dichotomously (1-city, 2-village).

The individual conditions included:

- School achievement was measured subjectively on a one-item visual scale based on E. Goodman's concept [14]. The scale takes the form of a ladder, where ‘0’ represents the worst academic performance and ‘10’ represents the best academic performance compared to other students in the class.
- Personal values were measured on a scale derived from the spirituality tool developed by HBSC members [15]. Five out of ten questions of this scale were selected, which create an internally coherent factor (Cronbach’s coefficient 0.795). The young people answered how important they feel the following items are: the meaning of life, a positive attitude towards others, and care for the natural environment (five possible answers, ranging from *completely unimportant* to *very important*). The range of the abbreviated scale is 0–20 points, and its structure is single-factor according to the PCA. The full Polish version of the spirituality scale can be found in the appendix along with marked selected questions.
- Current involvement in adolescent groups, as measured on a four-point frequency scale, ranging from 1 (*never or almost never*) to 4 (*daily or almost daily*). How often young people participate in such groups was asked, giving examples of scouting, sports associations and religious organisations.

The following socio-economic conditions were considered:

- The FAS (Family Affluence Scale). It is a tool developed and constantly modified by the members of the HBSC network [16]. In the current version (sometimes referred to as the FASIII) it consists of six questions and ranges from 0–13 points.
- Subjective assessment of the family social position. There is a one-item visual scale with a structure similar to the scale of academic achievement.
- Attitude towards school. It is a single question assessing how much students like their school, used in the HBSC research as a compulsory item from the beginning of their existence (1985/86).
- The scale of social ties (social capital) in the neighbourhood [17]. It consists of four statements and ranges from 0–16 points. The youth assessed on a 5-point scale how much they agreed with the statements given. This scale has a univariate structure and reliability at Cronbach’s alpha level of 0.712.

### Statistical analysis

For the purposes of some analyses, the independent variables have been categorised. The division criteria known from the literature were used, or it was assumed that the mean level contains 50% to 60% of the middle values. The distribution of the values of the independent variables is presented in the Tables, which provide also the characteristics of the sample. The level of missing data for independent variables was small, with a maximum of 90 cases.

The EECII values are presented as mean with the SD standard deviation. Non-parametric tests, such as the Mann-Whitney or Kruskal-Wallis test, were used for comparisons of different groups of adolescents.

A multivariate analysis was also performed, estimating the general linear model (GLM) with 2-way interactions. Due to the lack of data, the model could be estimated only for 94% of cases (N = 4,665).

## Results

In the study group, students obtained a mean EECII score of 5.59 (SD = 2.65). It is 44.9% of the maximum possible score (12 points). The median is lower than the mean (5.00), which confirms the deviation from the normal distribution (p <0.001 in the Kolmogorov-Smirnov test). Taking into account individual questions, the intentions of participating in volunteering were rated the worst, and the planned involvement in matters affecting the local community was rated the highest. Every fifth respondent (20.6%) definitely did not plan any prosocial activities, providing 1–2 answers to all three questions.

Taking into account the demographic characteristics (Table 1), stronger intentions of prosocial activities were demonstrated among girls than among boys. A systematic decrease in the EECII index was observed in the subsequent age groups (p = 0.002). The correlation with the place of residence turned out to be statistically insignificant, with a tendency towards a more positive assessment of intentions in rural areas. However, it was shown that residence modifies the relationship between gender and the EECII levels. In the group of boys, the analysed mean index remains at a similar level in cities and in rural areas (5.10 ± 2.58 vs. 5.12 ± 2.55). In the group of girls, schoolgirls from cities obtained worse results than schoolgirls from rural areas (5.54 ± 2.69 vs. 5.74 ± 2.71). Therefore, gender differences are increasing in rural areas. The relationship with the level of family affluence was statistically significant. Young people from more affluent families more often declared their willingness to participate in activities for the benefit of others.

**Table 1:** The mean EECII index by demographic characteristics and family affluence

Variable	N (%)	EECII level		Group comparison	
		Mean	SD	Test	p
Gender					
Boys	2220(44.7)	5.11	2.57	Z=-6.50	<0.001
Girls	2752(55.3)	5.62	2.70		
Age					
13 yrs	1835(36.9)	5.52	2.60		
15 yrs	1697(34.1)	5.39	2.70	H(2)=12.21	0.002
17 yrs	1440(29.0)	5.23	2.66		
Place of living					
Urban areas	2998(60.3)	5.34	2.65	Z=-1.48	0.139
Rural areas	1959(39.4)	5.46	2.66		
Missing values	15(0.3)				
FAS level					
Low	1455(29.3)	5.26	2.58		
Average	2268(45.6)	5.35	2.63	H(2)=7.640	0.022
High	1159(23.3)	5.59	2.73		
Missing values	90(1.8)				
Family social position					
Low	931(18.7)	5.16	2.58		
Average	2852(57.4)	5.29	2.55	H(2)=40.02	<0.001
High	1148(23.1)	5.86	2.88		
Missing values	41(0.8)				

FAS – Family Afflluence Scale; EECII - Expectations for Engagement in Community Issues Index; Z - Manna-Whitney nonparametric test; H - Kruskal-Wallis nonparametric test, degrees of freedom (d.f.) given in the parenthesis

In a similar way, Table 2 shows the mean EECII indexes in groups differing in selected individual and social characteristics. All five traits showed a very strong correlation with the intention to engage in prosocial activities after finishing high school. The strongest seems to be the link between the positive value system and the current involvement in youth groups.

**Table 2:** The mean EECII index by selected individual characteristics, the social capital of the school and the social ties in the area

Variable	N (%)	EECII level		Group comparison	
		Mean	SD	Test	p
School achievements					
Poor Average	889(17.9) 2671(53.7)	5.18 5.31	2.66 2.59	H(2)=21,45	<0.001
Good	1380(27.8)	5.66	2.74		
Missing values	32(0.6)				
Personal values level					
Low	923(18.6)	4.54	2.39		
Average	2866(57.6)	5.28	2.51	H(2)=219,97	<0.001
High	1136(22.8)	6.37	2.91		
Missing values	48(1.0)				
Current youth group engagement					
Never or hardly ever	3905(78.5)	5.19	2.62		
Monthly	429(8.6)	5.83	3.40	H(3)=120,85	<0.001
Weekly	399(8.0)	6.21	2.68		
Daily or almost daily	169(3.4)	6.92	3.05		
Missing values	70(1.4)				
Neighbourhood social capital					
Low	1203(24.2)	4.97	2.62		
Average	2418(48.6)	5.33	2.54	H(2)=72,70	<0.001
High	1311(26.4)	5.87	2.83		
Missing values	40(0.8)				
Liking school					
Don’t like it at all	487(9.8)	5.11	2.70		
Don’t like it very much	978(19.7)	5.05	2.59	H(3)=46,14	<0.001
Like it a bit	2127(42.8)	5.38	2.62		
Like it a lot	1376(27.7)	5.74	2.69		
Missing values	4(0.1)				

EECII, Expectations for Engagement in Community Issues Index; H - Kruskal-Wallis nonparametric test, degrees of freedom (d.f.) given in the parenthesis

The presented results show that, in simple comparisons, of the ten analysed factors only one (place of residence) did not show a significant relationship with the EECII. In order to identify independent predictors of this index’s variability, a general linear model was estimated, which allows assessment of the isolated effect of individual variables and the interactions between the analysed factors. In the first step, the significance of all possible 2-way interactions was checked. The second step was limited to statistically significant interactions. The main effect of gender (p <0.001), age (p = 0.024), family social standing (p = 0.001), represented values (p <0.001), perception of the social capital of the neighbourhood (p <0.001), attitude to school (p = 0.001) and participation in youth groups (p <0.001) turned out to be significant. Moreover, family social standing independently influenced the EECII variability in interaction with family affluence (p = 0.004) and gender (p = 0.006). The interaction between the place of residence and academic achievement (p = 0.022) as well as gender and attitude to school (p = 0.043) were also significant. Introducing the interaction between participation in adolescent groups and age into the model improves the quality of the model, although this interaction is on the verge of statistical significance (p = 0.060). Without it, the main effect of age is negligible.

**Figure 2:**
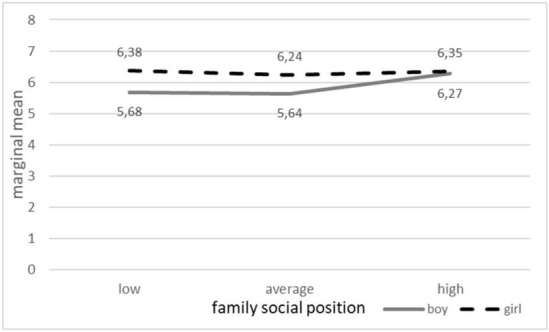
The mean EECII depending on gender and family social position

Figure 2 shows the combined effect of gender and family social standing as an example of an interaction. These are the theoretical levels of the EECII mean after adjusting for the other analysed factors. In the group of girls, the family social standing does not differentiate the EECII values. In the group of boys, significantly higher EECII values are recorded in the most privileged group of families, where the gender differences disappear.

A detailed analysis of the other significant interactions mentioned above indicates groups of adolescents where the interest in prosocial activities decreases despite the improvement of one of the analysed factors. For example, in the most affluent families, improving the social position of the family is correlated with a decline in EECII. In poor and moderately affluent families, the improvement of the social position favours the growth of the EECII. In turn, the improvement in academic achievement is associated with a decline in the EECII in rural areas and an increase in the value of this index in cities.

## Discussion

The above article is the first in a series which presents the results of HBSC 2018 studies related to the participation of young people in social life. The considerations are based on the hypothesis that it is one of the developmental assets which ought to be used in health promotion programs [18]. Empirical studies used a short scale of intentions to take up prosocial activities, which in the light of knowledge available to us has not been tested on the Polish population so far. Many authors emphasised that in studies dealing with similar issues (including those on volunteerism) the studied group mainly consisted of adults [19]. It is worthwhile to discuss the obtained results on the background of previous studies as well as in the context of legal and social determinants, such as the attitude towards activities for the benefit of the local community prevailing in our country.

The results indicate that Polish youth express moderate interest in prosocial activities after graduation from secondary school, but that one in five teenagers in fact never considered taking up such activity. Nonetheless a certain improvement may be observed, as in 2013 only one in ten secondary school students mentioned being useful for others as one of his or her important objectives in life [20].

The presented results are confirmed in earlier studies, where gender, apart from other demographic factors, affects the existence of prosocial attitudes and the willingness to become involved in social life [21, 22, 23], although the observed direction of such influence was unequivocal. Studies conducted in the U.S. have demonstrated that females were more willing to take up voluntary activities [21], as opposed to Spain, where males were more likely to become volunteers than females [22]. On the other hand, studies of young people in China have not shown any difference between the genders in declaring altruistic behaviours [24]. In Poland studies consistently indicate that girls are more committed to volunteering [25, 26], which was confirmed by the presented HBSC 2018 results. Greater motivation among girls to get involved in prosocial behaviours may be a result of socialisation of boys and girls in our cultural zone [21, 27]. Whether or not the intentions to take up social activities declared by teenage girls will be reflected later in life is another issue. On the basis of studies among adults it was found that men are more socially engaged in comparison to women [28, 29], albeit not necessarily in activities of charitable nature [30].

Our analyses have shown that among considered individual and environmental features the intention to engage in prosocial activities is most predominantly associated with a positive system of values, which we assess on the spirituality scale, and with involvement in youth groups [15]. In the light of international comparisons, the significance of spirituality and positive values in life is significantly more often underlined by girls than by boys, which may also translate into a higher level of motivation for prosocial behaviour in this group [15, 31, 32]. In particular girls ascribe more importance than boys to the values which refer to relationships with others [15]. Polite and kind behaviour towards other people is highly valued by girls regardless of age, while the assessment of ‘being compassionate/forgiving’ declines with age, regardless of gender. Decline in the importance of positive values among students with age, presumably resulting from social factors or cognitive processes and teenage rebellion [21, 27], may partly account for the results we obtained, which indicate that the intentions to take up prosocial activities decline among older adolescents. The results of U.K. studies indicating that after the decline in social activity among teenagers it then appears to rise among university students seems to confirm this hypothesis [22, 33].

The observed positive interaction of the EECII scale with engagement in social organisations is reflected in studies which indicate that people who volunteer in their youth also volunteer as adults at a higher than average rate [34]. The positive impact of the frequency of involvement in social organisations on prosocial behaviours has also been observed in China [24]. Further, the studies conducted in Poland in the Jezioro Rożnowskie Communes Association have shown that youth involvement in the activities of social organisations prepares for active participation in social life when the following conditions are fulfilled: social organizations are given an opportunity to act; charismatic leaders are present; and local authorities have favourable attitudes, which enables true involvement in the social life of the community [35].

It is worthwhile to nurture the conditions which consolidate the involvement of adolescents in constructive leisure time activities, as this makes them less likely to take up risky behaviours [25].

When analysing the decline of the EECII index with students’ age, the unique legal conditions in Poland cannot be overlooked. Achievements in the area of social activity are a part of special achievements listed on the school graduation report, for which additional points may be scored during the recruitment process for higher education3Regulation on the recruitment to secondary schools: Rozporządzenie Ministra Edukacji Narodowej z dnia 14 marca 2017 r. w sprawie przeprowadzania postępowania rekrutacyjnego oraz postępowania uzupełniającego na lata szkolne 2017/2018–2019/2020 do trzyletniego liceum ogólnokształcącego, czteroletniego technikum i branżowej szkoły I stopnia, dla kandydatów będących absolwentami dotychczasowego gimnazjum (Dz. U. z 2017 poz. 586). It may be supposed that some young respondents had this extra motivation to take up such activities, in the belief that they would be continued in the future. This is an example of how organisational determinants affect the level of individual values. On the other hand, a utilitarian decision to volunteer made under this kind of pressure distorts the very concept of volunteer work (voluntariness), and as such does not enhance permanent attitudes which could play a protective role in the future [36].

Planned social engagement also depends on health assets shaped at the social level, in particular the support of family and school. Parental attitudes and the atmosphere in the family play a decisive role in shaping prosocial behaviours [37, 38]. Volunteer work by a person below 18 years of age requires the consent of his or her legal guardian, so parents’ approval for this type of activity is a must. Study results show a strong impact of the parents’ personal example, especially in the case of younger children [22], and the unwillingness of parents to engage in volunteer work frequently causes young people to also be unwilling to do so [37]. An important

factor which has a positive impact on volunteering is a friendly, ‘democratic’ atmosphere in the family [39]. On the other hand, young people are relatively unlikely to decide to take up prosocial activities on the encouragement of family members. Apart from the importance of mental support, the HBSC study has shown, as in Spain [22] and the U.S. [40], that higher socio-economic status of the family increases volunteerism.

Introducing young people into the world of values, shaping their openness to the world and other people, and encouraging them to actively participate in social life are key educational objectives of the school environment [39]. This includes building motivation to take up volunteer work. Both teachers as well as parents engaged in the activities of the Parent’s Council recognise the importance of volunteering as a protective factor and include it in their preventive and educational programs. As in the family, the effectiveness of such initiatives and the problems encountered in young people’s involvement may be caused by the weak implementation level of empowerment. Increasing the autonomy of young people and providing them a choice of the type of activities they may wish to take up is an important issue [8].

This study has some limitations. Despite the fact that approximately 10 factors which may support prosocial attitudes have been taken into account, their selection depended on the range of subjects covered by the HBSC questionnaire. It is also difficult to draw conclusions about the motivation for taking up activities for the community, and the existing barriers. It would be worthwhile to broaden the list of potential determinants of prosocial attitudes in subsequent publications and review whether favourable results in the EECII scale are related to a higher assessment of psycho-social health. It is also advisable to start cooperation with other countries of the HBSC network, where the same scale was included in the 2018 studies.

Despite the abovementioned flaws the study has many advantages. First, it concentrates on a young population greatly diversified in terms of age, which is a much less frequent object of volunteerism studies than are adults [19]. Second, testing a short scale which measures the intentions to engage in prosocial activity is a strength of the study. Third, the conducted research has a number of practical implications. It would be worth strengthening the involvement of young people in community activities, taking into account the specifics of different demographic groups, personal characteristics, and young people's perceptions of social relations in their surroundings. We hope that this publication will initiate a series of papers based on HBSC data using this scale, and in the future it will be possible to present Polish data on the backdrop of other countries and to demonstrate the relationship between the attitude towards prosocial activity and indicators of psychosocial health.

## Appendix

**Polish version of spirituality scale for adolescents [HBSC Research Protocol 2017/18 ]**

Jak bardzo ważne jest dla Ciebie…. / *How important is it for you to…*

1. Być miłym dla innych ludzi **/** *Be kind to other people**
2. Być wyrozumiałym dla innych **/** *Be forgiving of other people**
3. Okazywać szacunek innym / *Show respect for other people**
4. Czuć, że życie ma cel i sens / *Feel that your life has meaning or purpose**
5. Doświadczać radości w życiu */ Experience joy in life*
6. Odczuwać więź z naturą lub przyrodą / *Feel connected to nature or wilderness*
7. Troszczyć się o środowisko naturalne / *Care for the natural world**
8. Modlić się lub medytować / *Meditate or pray*
9. Czuć związek z siłą wyższą rządzącą światem / *Feel connection to a higher spiritual power*
10. Czuć przynależność do czegoś większego niż ja sam */ Feel a sense of belonging to something greater than yourself*

Kategorie odpowiedzi */ Response categories*: 1- wcale nie jest ważne */ not at all important;* 5- jest bardzo ważne */ very important*

*Five items included in the personal values index correspond to others (1–3), self (4) and nature (7) domains.
